# C*-BODIPYs: Exploring a New Strategy to Transfer Chirality towards BODIPY Chiroptics^†^

**DOI:** 10.3390/ecsoc-23-06610

**Published:** 2020-01-20

**Authors:** Josué Jiménez, Juan Sánchez-Camacho, Florencio Moreno, Antonia R. Agarrabeitia, Teresa Arbeloa, Trevor A. Cabreros, Gilles Muller, Jorge Bañuelos, Beatriz L. Maroto, Santiago de la Moya

**Affiliations:** 1 Departamento de Química Orgánica, Facultad de Ciencias Químicas, Universidad Complutense de Madrid, Ciudad Universitaria s/n, 28040 Madrid, Spain;; 2 Departamento de Química Física, Facultad de Ciencias y Tecnología, Universidad del País Vasco-EHU, 48080 Bilbao, Spain;; 3 Department of Chemistry, San José State University, San José, CA 95192-0101, USA;

**Keywords:** BODIPYs, circular dichroism, circularly polarized luminescence, CPL-SOMs

## Abstract

C*-BODIPYs, that is, boron dipyrromethenes (BODIPYs) which have chiral carbons attached directly to the boron center, are introduced for the first time. These novel chiral BODIPYs mean a new strategy for the chiral perturbation of the inherently achiral BODIPY chromophore that is directed to enable chiroptical properties. Their preparation is very simple and only implies the complexation of a dipyrrin with an enantiopure dialkylborane having boron bonded to chiral carbons.

## 1. Introduction

Circularly polarized luminescence (CPL) is a chiroptical phenomenon consisting of the differential emission of right- and left-handed circularly polarized light by chiral luminescent systems, such as molecules, ionic pairs, metal complexes, polymers, and supramolecular aggregates among others [1]. Interest in CPL is due to the higher resolution provided by the circular polarization of the light and application has been found for the improvement and potential development of multiple photonic tools, such as display devices including three-dimensional (3D) optical displays, optical storage, and processing systems; spintronics-based devices; biological probes and signatures; security tags; CPL lasers; enantioselective CPL sensors; and light-emission systems for asymmetric photosynthesis. In addition, CPL is a valuable source of information in chiral emitting excited states (CPL spectroscopy) and it can also be used to discern and quantify chiral enantiomers (CPL chiral sensing) [2].

The level of CPL is quantified by the luminescence dissymmetry factor (*g*_lum_), which lies between −2 and +2 (completely right- and left-circularly polarized emission, respectively). The highest levels of *g*_lum_ have been found for photoluminescent lanthanide coordination complexes (| *g*_*lum*_ | into the range of 0.05–0.5) [1]. However, the emission efficiency of these systems is usually small, due to the nature of the electronic transitions involved in the luminesce phenomenon. Other systems showing moderated values of *g*_lum_ are some helical polymers or supramolecular aggregates based on organic chromophores (| *g*_*lum*_ | typically into the range of 10^−3^–10^−1^) [3], but they also present low emission efficiencies as a consequence of the hierarchical aggregation.

In this sense, simple organic molecules (small, non-polymeric and non-aggregated) enabling CPL (CPL-SOMs) have recently attracted considerable attention due to their properties associated with their small size and organic nature (organic-solvent solubility and ease of manufacturing processes), and with their advantageous photophysical properties (high emission efficiencies and possibility of modulating photophysical signatures by structural variations) [2,4]. However, CPL-SOMs exhibit small levels of | *g*_*lum*_ | (typically in the range of 10^−5^–10^−3^). Consequently, despite their advantages, CPL-SOMs are rare, and they are restricted to a few structural designs, which usually involve long and inefficient synthetic routes. Therefore, the development of new, efficient CPL-SOMs is a priority objective in CPL research [2].

**Figure 1.**
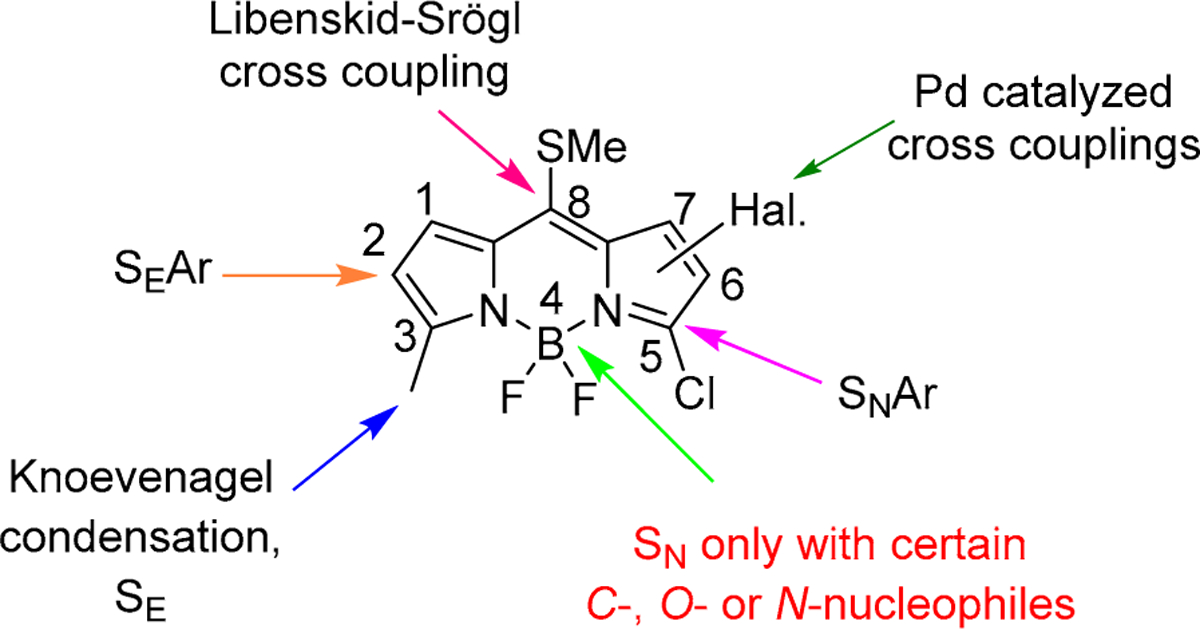
Some useful chemical transformations in BODIPY dyes. In red, functionalization at the boron atom reported to date.

BODIPYs (boron dipyrromethenes or 4-bora-3a,4a-diaza-*s*-indacenes) (see Figure 1) are ideal chromophores with unique and tunable photophysical properties. Thus, BODIPY chromophores usually emit a relatively sharp fluorescent peak with high fluorescence quantum yield and a high molar absorption coefficient [5]. They also have good solubility, chemical robustness, thermal and photochemical stability, and are one of the few structures that can fluoresce over the entire visible and in the near IR electromagnetic spectrum [5,6]. But the striking success of these dyes relies, not only in their excellent properties, but mainly on the chemical versatility of their chromophoric core, amenable to a wide range of chemical modifications [7,8]. In fact, recent advances in BODIPY chemistry have boosted the post-functionalization of easily accessible parent chromophores, and therefore have afforded a wide pool of multifunctional and tailor-made dyes for specific photonic applications [5,6]. Figure 1 shows the most popular BODIPY transformations in different positions of the BODIPY core. In this regard, the boron atom is a key position for functionalization of BODIPYs keeping the photophysical properties of the chromophore. Therefore, it has led to easy preparation of dyes with enhanced photostability for lasing, improved water solubility for biological applications, boosted energy-transfer processes for collecting light efficiently, among other valuable applications [7–10].

**Figure 2.**
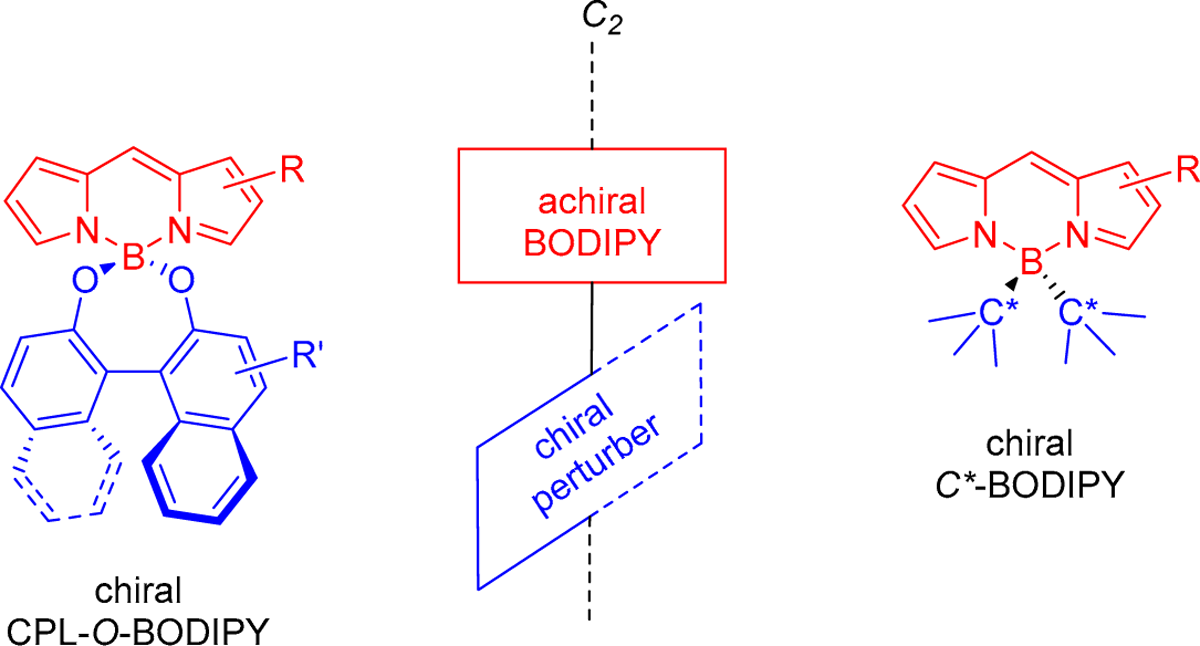
Chiral CPL-*O*-BODIPY vs. chiral C*-BODIPY.

BODIPY functionalization at the boron atom has also been used to chirally perturb the inherently achiral BODIPY chromophore towards CPL. In this context, our research group introduced a new structural design for CPL-SOMs, where an achiral BODIPY chromophore is chirally perturbed by orthogonally tethering a single 1,1′-binaphthyl moiety to it by functionalization at the boron atom through oxygen bridges (see CPL *O*-BODIPY in Figure 2) [13]. The chiral perturbation of the BODIPY chromophore, which comes from the axial chirality of the 1,1′-binaphthyl moiety, provides a |*g*_lum_| value of 1 × 10^−3^. This is a small value for *g*_lum_, as is the case for most SOMs, and therefore there is still room for improvement in this novel design.

We hypothesize that a more efficient chiral perturbation over the chromophore could be achieved by bringing the chiral perturber closer to the BODIPY core. One strategy for doing so could be to attach chiral carbons directly to the boron atom, in other words, building C*-BODIPYs (see C*-BODIPY in Figure 2). However, the synthesis of C*-BODIPYs is not trivial. In fact, it has not been reported to date. *C*-BODIPYs involving *C*_sp3_ atoms directly bonded to the boron center are rare [14] probably due to the lower stability of the boron chelate as compared with other *C*-BODIPYs or with *F*-BODIPYs (note the lower Lewis acid character of the involved dialkylboron moiety as compared with dialkynylboron, diarylboron, or especially, difluoroboron).

## 2. Results and Discussion

### 2.1. Synthetic Development

**Scheme 1.**
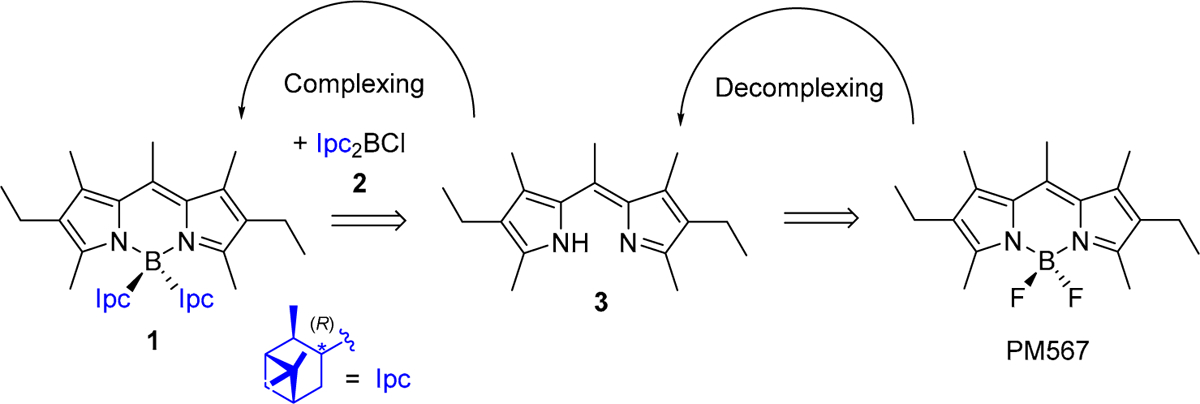
Proposed synthetic plan for C*-BODIPY **1** based on isopinocampheyl (I_pc_).

The synthetic route towards C*-BODIPYs was designed in two steps (Scheme 1) as follows: (1) Decomplexation of a commercial *F*-BODIPY to obtain the corresponding dipyrrin **3**, following the methodology developed by us [15] and (2) complexation of the dipyrrin **3** with an enantiopure dialkylboron chloride [14] having chiral carbons attached to the boron. To test the workability of the proposed synthetic route, we chose 2,6-diethyl-1,3,5,7,8-pentamethylBODIPY (PM567) as starting *F*-BODIPY, because of its commercial availability and because of its electron richness (due to polyalkylation), which would stabilize the formed C*-BODIPY. As the enantiopure dialkylboron source, we chose a derivative of a natural product from the *Chiral Pool*, *B*-chlorodiisopinocampheylborane (I_pc2_BCl, **2**), which is also commercially available.

**Scheme 2.**
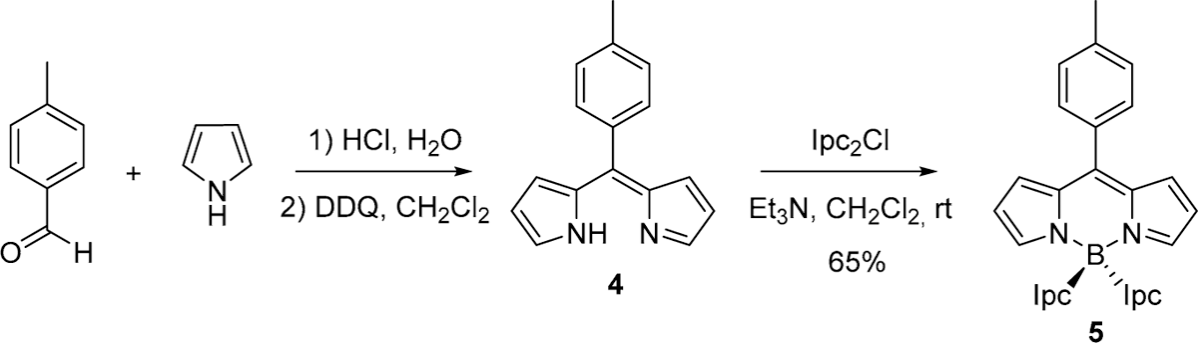
Preparation of the first C*-BODIPY.

However, the reaction of the dipyrrin **3** (obtained by treatment of PM567 with trifluoromethanesulfonic acid [15]) with I_pc2_BCl (**2**) did not lead to the expected **1**. The reaction was unsuccessful probably due to steric reasons because of the high volume of the I_pc_ moieties. Therefore, we decided to use a dipyrrin without substituents at positions 3,5 (α to the nitrogen), to diminish the sterical hindrance near the boron-complex site (see **4** in Scheme 2). Since the *F*-BODIPY precursor of dipyrrin **4** is not commercially available, we prepared the required **4** from the corresponding aldehyde and pyrrole, following a standard procedure used in synthesis of *F*-BODIPY (see Scheme 2) [16]. Having **4** in hand, its reaction with I_pc2_BCl (**2**) in the presence of triethylamine finally led to C*-BODIPY **5** with a 65% yield.

### 2.2. Photophysical Study

**Figure 3.**
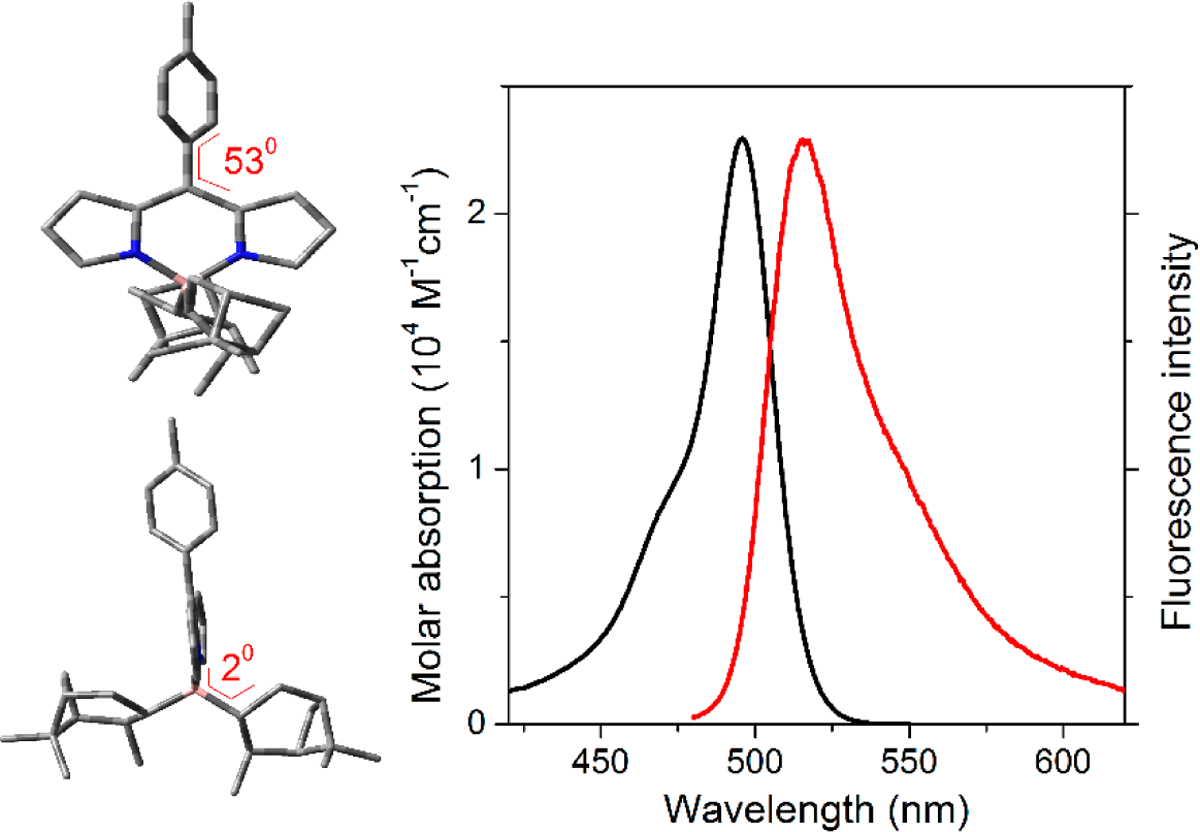
Absorption (**black**) and normalized fluorescence (**red**) spectra of **5** in diluted solution (2 mM) of methanol. The corresponding optimized ground state geometry (PCM-B3PYP/6–31g* in methanol) is also added (**top** in a front view and **bottom** in a side view), together with key dihedral angles to highlight the orientation of the rings appended at 8-position and at the boron bridge.

The spectroscopic signatures of **5** feature an absorption band centered at around 495 to 505 nm, depending on the solvent properties (Figure 3). The corresponding excitation yields a fluorescence emission located at 515 to 520 nm (Figure 3). These spectral band positions match those recorded for the corresponding *F*-BODIPY bearing 8-tolyl [17], and even for the simplest unsubstituted *F*-BODIPY (BDP) [5]. From this comparison, we can assume that, on the one hand, the pendant functionalization at the boron atom does not affect the spectral shift. Such a trend could be expected since the boron acts as a bridge to infer rigidity to the whole dipyrrin backbone and it does not take part in the delocalized π-system. On the other hand, at least in the ground state, the 8-phenyl is electronically decoupled with the dipyrrin π-conjugated system. Indeed, the theoretically minimized ground state geometry predicts that the phenyl ring is twisted around 53° (Figure 3), owing to the steric hindrance with the adjacent hydrogens, avoiding any resonant interaction, which will promote pronounced spectral red shifts.

Regarding the probability of the electronic transitions, the molar absorption of **5** is lower than that typically registered for *F*-BODIPYs [5] but reasonably high (up to 23,000 M^−1^cm^−1^ in Table 1). However, the fluorescence emission is very weak (lower than 4%), with a multiexponential decay curve dominated by fast lifetimes (lower than 1 ns, Table 1). A similar fluorescence efficiency is attained for the corresponding *F*-BODIPY counterpart [17,18] and it was attributed to the phenyl free motion at the key 8-position. Such conformational freedom, especially upon excitation, enhances the non-radiative deactivation channels related to internal conversion. Moreover, the excited state dynamics of the 8-phenyl-*F*-BODIPY has been the subject of a theoretical simulation to unravel it [19]. The computational study reveals that upon excitation a metastable dark state can be populated from the locally excited state. In this non-fluorescent state, the geometry is drastically distorted. The 8-phenyl is coplanar with the dipyrrin core and electronically coupled, leading to a marked butterfly-like distortion of the chromophore along the transversal axis. In fact, the optimized (TD-PCM- B3LYP/6–31g*) first excited state geometry (locally excited state) anticipates an interaction between the 8-phenyl and the dipyrromethene, since the 8-phenyl twisting dihedral angle decreases from 53° in S_0_ down to 43° in S_1_.

Another additional source of nonradiative relaxation can be related to the accommodation of the bulky chiral rings appended to the boron atom. It has been previously reported that constrained and sterically strained functionalization at this position could lead to pronounced planarity distortions or bending of the chromophore, with a detrimental impact in the fluorescence response [14]. However, in the case of **5**, the chromophore seems to retain its planarity, and both pendant chiral rings are disposed almost perpendicular up and down the dipyrromethene plane according to the theoretical calculations (Figure 3). Excited state calculations suggest that the orthogonal disposition remains the same upon excitation. Moreover, the fluorescence efficiencies recorded for this C*-BODIPY (Table 1) are similar to those reported for its *F*-BODIPY counterpart [17] suggesting that the bulky rings at the boron atom are conformationally locked, and hence the nonradiative deactivation channels are mainly ruled by the aryl free motion around the 8-position.

### 2.3. Chiroptical Behaviour

The absolute value of the specific optical rotation of new C*-BODIPY **5** ([α]D^20^ +402.0 (*c* 0.10 CHCl3)) was much lower than those recorded for binaphthyl-*O*-BODIPYs (for example, ([α]D^20^ −5076.2 (*c* 0.12, CHCl3) for *O*-BODIPY based on PM567 and BINOL [13]). Nevertheless, we did detect a circular dichroism (CD) signal (CHCl_3_, 4.6 × 10^−5^ M) at its vis absorption maximum, whose *g*_abs_ value (|*g*abs| 0.6 × 10^−3^) fell in the same range as said *O*-BODIPY (|*g*_abs_| ~1.0 × 10^−3^). Moreover, despite the extremely low fluorescence of **5**, we were able to record its CPL spectrum (*c*-hexane, 2 × 10^−3^ M), showing a maximum matching the vis emission maximum, with a |*g*_lum_| value of 0.7 × 10^−3^, which is also comparable to the |*g*_lum_| value exhibited by said *O*-BODIPY (0.7 × 10^−3^).

These results show that the chiral perturbation exerted over the BODIPY chromophore by the chiral C_sp3_ centers directly attached to the boron in **5** is similar to that exerted by the binaphthyl unit, which is farther away from the chromophore. Therefore, placing the chirally perturber closer to the chromophore is not enough to achieve a higher chiroptical response, but other features need to be taking into account. For example, the conformational freedom of the alkyl moieties in **5**, as compared with the rigidity of the spiranic binaphthyl unit in said *O*-BODIPY can be one of the features to be taken into account.

## 3. Conclusions

For the first time, chiral carbons have been introduced at the boron atom of a BODIPY to obtain the first C*-BODIPY. It can be easily obtained by complexation of a dipyrrin with an enantiopure dialkylborane having boron bonded to chiral carbons. This C*-BODIPY has been designed as a proof of concept that this can be a new strategy for the chirality transfer to the BODIPY chromophore, directed towards chiroptics. Indeed, the recorded CD and CPL spectra demonstrate that the chirality transfer is indeed taking place. Although the almost inexistent fluorescence of this new BODIPY makes it unsuitable as a CPL enabling molecule, this new functionalization is a strategy to explore for CD applications. Further research is in progress which is directed to improve both fluorescence efficiency and CD and CPL activities.

## 4. Materials and Methods

### 4.1. Synthetic Procedures

#### General.

Common solvents were dried and distilled by standard procedures. All starting materials and reagents were obtained commercially and used without further purifications. Elution flash chromatography was conducted on silica gel (230 to 400 mesh ASTM). Thin layer chromatography (TLC) was performed on silica gel plates (silica gel 60 F254, supported on aluminum). The NMR spectra were recorded at 20 °C, and the residual solvent peaks were used as internal standards. The NMR signals are given in ppm. The DEPT-135 NMR experiments were used for the assignation of the type of carbon nucleus (C, CH, CH_2_, and CH_3_). The FTIR spectra were recorded from neat samples using ATR technique and IR bands are given in cm^−1^. Optical rotations in chloroform solution (dye concentration, c, expressed in g/100 mL) were recorded at 293 K on an Anton Paar MCP 100 polarimeter.

#### Synthesis of C*-BODIPY 5.

A solution of **4** (22 mg, 0.094 mmol), triethylamine (47 mg, 0.47 mmol) and I_pc2_Cl (151 mg, 0.47 mmol) in CH_2_Cl_2_ (5 mL) under argon, was reacted at room temperature for 24 h. Then, water (10 mL) was added, the phases were separated, and the aqueous phase was extracted with CH_2_Cl_2_ (3 × 5 mL). The combined organic extracts were washed with water (5 mL), dried over Na2SO4 and filtered. The solvent was evaporated under reduced pressure. The crude was purified by column chromatography on silica gel, using hexane as the eluent. **5**: 31 mg (63%). Orange solid. M.p. > 85 °C (decomposes). Rf = 0.51 (hexane). [α]^D^_20_ +402.0 (*c* 0.10 CHCl_3_). ^1^H NMR (acetone-*d6*, 300 MHz) δ 8.18 (s, 2H); 7.52 (d, *J* = 8. 2 Hz, 2H); 7.42 (d, *J* = 7.8 Hz, 2H); 6.94 (dd, *J* = 4.3, 1.2 Hz, 2H); 6.64 (dd, *J* = 4.3, 1.8 Hz, 2H); 2.47 (s, 3H); 2.39 (m, 2H); 2.27 (ddd, *J* = 14.0, 7.4, 2.7 Hz, 2H); 2.03 to1.99 (m, 1H); 1.92 (m, 2H); 1.55 (m, 2H); 1.38 (td, *J* = 5.9, 2.0 Hz, 2H); 1.26 (td, *J* = 7.3, 2.0 Hz, 2H); 1.13 (s, 6H); 1.12 (s, 6H); 0.45 (d, *J* = 9.1 Hz, 2H); and 0.33 (d, *J* = 7.0 Hz, 6H) ppm. ^13^C NMR (acetone-*d6*, 75 MHz) δ 148.5 (C); 144.0 (CH); 141.3 (C); 135.5 (C); 133.0 (C); 131.2 (CH); 129.8 (CH); 128.4 (CH); 117.7 (CH); 50.9 (CH); 43.1 (CH); 40.2 (C); 40.0 (CH); 33.8 (CH_2_); 32.0 (CH_2_); 28.7 (CH_3_); 23.9 (CH_3_); 23.0 (CH_3_); and 21.4 (CH_3_) ppm. ^11^B NMR (CDCl3, 160 MHz) δ 2.25 ppm. FTIR *v* 2891, 1553, 1412, 1384, 1358, 1254, 1061, and 1027 cm^−1^.

### 4.2. Photophysical Signatures

Diluted dye solutions (around 2 × 10^−6^ M) were prepared by adding the corresponding solvent (spectroscopic grade) to the residue from the adequate amount of a concentrated stock solution in acetone, after vacuum evaporation of this solvent. UV-Vis absorption and steady-state fluorescence were recorded on a Varian model CARY 4E spectrophotometer and an Edinburgh Instruments spectrofluorometer (model FLSP920), respectively, using 1 cm path length quartz cuvettes. The emission spectra were corrected from the monochromator wavelength dependence, the lamp profile and the photomultiplier sensitivity. Fluorescence quantum yields (*ϕ*) were calculated using commercial PM546 (*ϕ*^r^ = 0.85 in ethanol) as the reference. The values were corrected by the refractive index of the solvent. Radiative decay curves were registered with the time-correlated single-photon counting technique using the same spectrofluorometer (Edinburgh Instruments, model FL920, with picosecond time resolution). Fluorescence emission was monitored at the maximum emission wavelength after excitation at 470 nm by means of a diode laser (PicoQuant, model LDH470, respectively) with 150 ps full width at half maximum (FWHM) pulses. The fluorescence lifetime (τ) was obtained after the deconvolution of the instrumental response signal from the recorded decay curves by means of an iterative method. The goodness of the exponential fit was controlled by statistical parameters (chi-square) and the analysis of the residuals.

### 4.3. CD and CPL Measurements

CD spectra were recorded on a Jasco (model J-715) spectropolarimeter using standard quartz cells of 1 cm optical-path length in chloroform solution, at a dye concentration of 4.6 × 10^−6^ M. Circularly polarized luminescence (CPL) and total luminescence spectra were recorded at 295 K in degassed *c*-hexane solution at a dye concentration of ca. 2 mM, on an instrument described previously [20], operating in a differential photon-counting mode. The light source for excitation was a continuous wave 1000 W xenon arc lamp from a Spex Fluorolog-2 spectrofluorimeter, equipped with excitation and emission monochromators with dispersion of 4 nm/mm (SPEX, 1681B). To prevent artefacts associated with the presence of linear polarization in the emission [21], a high-quality linear polarizer was placed in the sample compartment and aligned so that the excitation beam was linearly polarized in the direction of emission detection (z-axis). The key feature of this geometry is that it ensures that the molecules that have been excited and that are subsequently emitting are isotropically distributed in the plane (x,y) perpendicular to the direction of emission detection. The optical system detection consisted of a focusing lens, long pass filter, and 0.22 m monochromator. The emitted light was detected by a cooled EMI-9558B photomultiplier tube operating in photo-counting mode.

### 4.4. Computational Methods

Ground and first excited states geometries were optimized at the density functional theory (DFT) level using the B3LYP hybrid method and the time dependent (TD-B3LYP) method, respectively. In both cases, the double valence basis set adding a polarization function (6 to 31g*) was used. The energy minimization was conducted without any geometrical restriction and the geometries were considered as energy minimum when the corresponding frequency analysis did not give any negative value. The solvent effect (methanol) was also simulated during the calculations by the self-consistent reaction field (SCRF) using the polarizable continuum model (PCM). All the theoretical calculations were carried out using the Gaussian 16 implemented in the computational cluster provided by the SGIker resources of the UPV/EHU.

## Figures and Tables

**Table 1.** Photophysical properties of C*-BODIPY **5** in diluted solution of apolar (cyclohexane) and polar (methanol) solvents.

	λab^1^ (nm)	10^−4^ · εmax^2^ (M^−1^cm^−1^)	λfl^3^ (nm)	ϕ^4^	τ^5^ (ns)
MeOH	496.0	2.3	515.5	0.037	0.37 (65%)–0.93 (31%)–4.43 (4%)
*c*-hexane	502.5	1.3	519.5	0.030	0.17 (71%)–0.67 (25%)–3.70 (4%)

1 Absorption wavelength,

2 molar absorption at the maximum (λab),

3 fluorescence wavelength,

4 fluorescence quantum yield,

5 lifetime.
